# Constructing a knowledge graph for open government data: the case of Nova Scotia disease datasets

**DOI:** 10.1186/s13326-023-00284-w

**Published:** 2023-04-18

**Authors:** Enayat Rajabi, Rishi Midha, Jairo Francisco de Souza

**Affiliations:** 1 Shannon School of Business, Cape Breton University, Grand Lake Dr., B1M 1A2 Sydney, Canada; 2 Department of Computer Science, Federal University of Juiz de Fora, Juiz de Fora, Brazil

**Keywords:** Open statistical data, Nova Scotia, Knowledge graph, Disease dataset

## Abstract

The majority of available datasets in open government data are statistical. They are widely published by various governments to be used by the public and data consumers. However, most open government data portals do not provide the five-star Linked Data standard datasets. The published datasets are isolated from one another while conceptually connected. This paper constructs a knowledge graph for the disease-related datasets of a Canadian government data portal, Nova Scotia Open Data. We leveraged the Semantic Web technologies to transform the disease-related datasets into Resource Description Framework (RDF) and enriched them with semantic rules. An RDF data model using the RDF Cube vocabulary was designed in this work to develop a graph that adheres to best practices and standards, allowing for expansion, modification and flexible re-use. The study also discusses the lessons learned during the cross-dimensional knowledge graph construction and integration of open statistical datasets from multiple sources.

## Introduction and motivation

The open government data movement has led to open data portals that provide a single point of access for a province or country. Open government data increases government transparency and accountability, contributes to economic growth and improves administrative processes [1]. This data is published hoping that different organizations’ data consumers can use it in the public and private sectors. A variety of published open datasets include multi-dimensional and statistical information such as census data, demographics, and public health data (e.g., number of disease cases) [2]. In itself, the data can be restrictive and not powerful enough to draw meaningful inferences. The datasets act as isolated pools of information that cannot be queried or linked. These sources are scattered in the government data portals, and users can access the information through specific searches in that data portal. The lack of formal semantics behind the open statistical data makes it impossible to form a network and link this kind of data to infer, create and query knowledge [3]. Interconnectivity between isolated datasets in open data gives a machine much information to work with, strengthening its ability to deduce relations and infer meaning. This study constructs a knowledge graph to connect various disease-related datasets in Nova Scotia Open Data (NSOD)^1^, a Canadian regional Open Data portal.

At time of this research, there are 11 provinces and territories in Canada with approximately 11,771 published datasets in different domains ranging from “Business and Economy” to “Health and Wellness” in various formats (e.g., CSV, JSON, and Excel) [4]. Most open datasets do not allow users to export data in RDF; the data are isolated while semantically linked. Hence, a human should manually connect various disease datasets and identify the diseases’ category to answer questions like: “Which viral diseases had the most cases in a province in 2017?”. 

This study intends to answer such questions using the Semantic Web technologies such as ontologies, RDF Cube as a multi-dimensional model, deductive reasoning rules, and generate a knowledge graph with semantic relationships. We link the instances of the disease-related datasets (metadata, dimensions, measures, and attributes) semantically on a schema level following the W3C standards and enrich them with a disease ontology. After constructing the knowledge graph, we pose a set of queries against the knowledge graph to demonstrate the power of the constructed knowledge graph over the interconnected datasets. The structure of this paper is as follows: Background section explains the background and the related studies in publishing datasets, particularly in the domain of multi-dimensional data models. Nova Scotia Open Data section describes the existing NSOD datasets. Methodology section presents the designed data model, ontology, and transformation process. Transformation challenges will be presented in Discussion section, followed by a Conclusion.

## Background

Statistical open data usually follows a multi-dimensional structure with dimensions and measures. Many studies have previously employed the RDF Data Cube vocabulary for statistical data [5, 6]. As an example, Escobar et al. [7] described the process of improving and enriching the quality of Barcelona’s official open data platform by employing multi-dimensional data and a linked open data assessment process. In another example, Klímek et al. [8] explained how the Czech Social Security Administration (CSSA) published its official pension statistics as Linked Oopen Data (LOD). They modeled the datasets using the Simple Knowledge Organization System (SKOS) vocabulary and the RDF Data Cube Vocabulary. The use of open statistical data in healthcare has also been used in the literature. As an example in healthcare, the PubMed knowledge graph [9] extracted over 29 million records from PubMed library and generated a knowledge graph to link bio-entities, authors, funding, affiliations, and articles. Similar previous studies, we followed the Linked Data standards and patterns [10, 11] and the Semantic Web protocol prescribed by W3C to construct a knowledge graph for a set of NSOD datasets.

## Nova Scotia Open Data

**Fig. 1:**
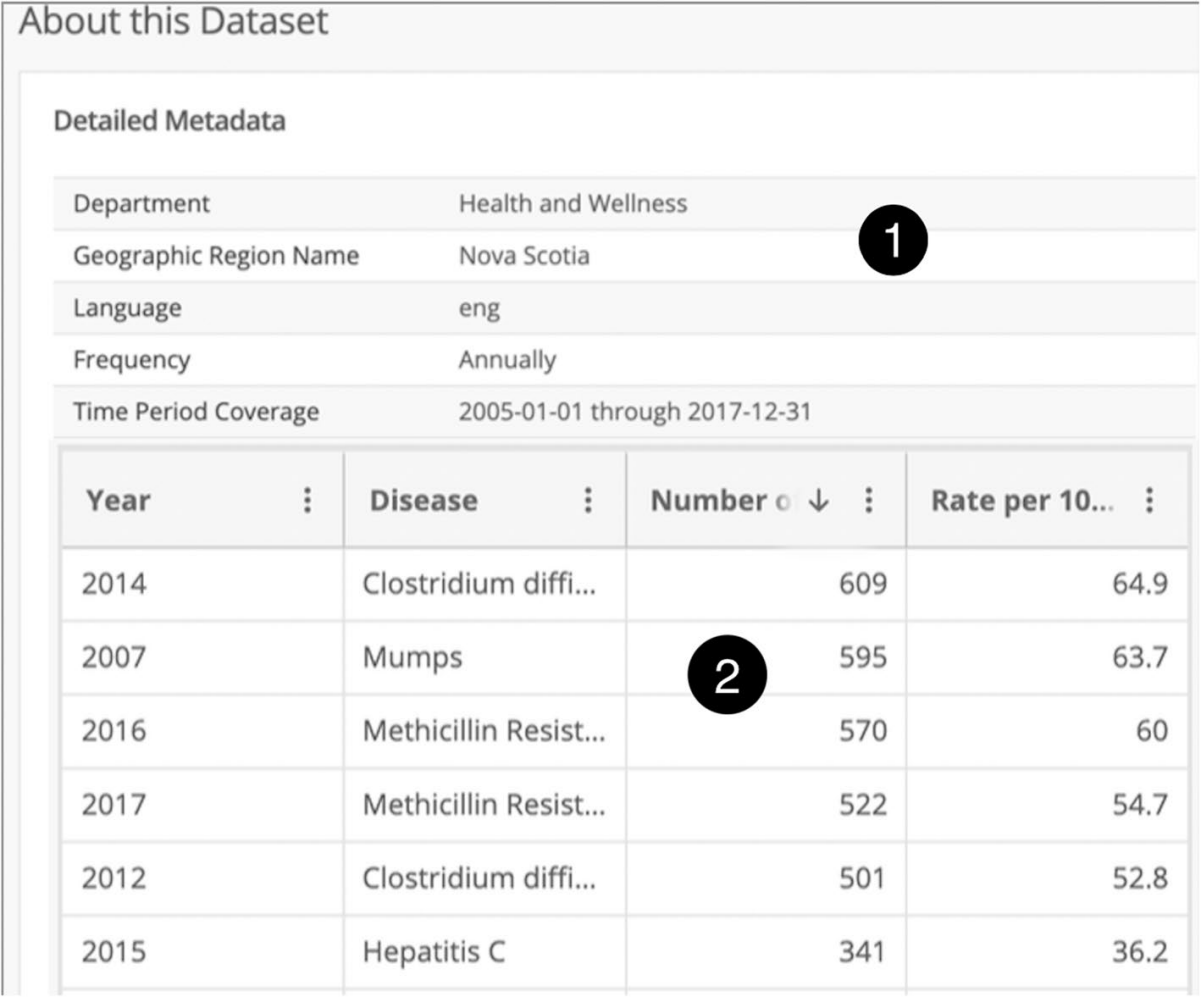
A disease dataset in the NSOD web portal (1: metadata, 2: observations)

Nova Scotia’s government has abundant resources in data and information collected and published in the NSOD web portal in the form of datasets. The main purpose of the NSOD portal is to allow individuals, particularly Nova Scotians in Canada, to efficiently access the information, understand their government, support their businesses, gain new insights, and make discoveries. The NSOD datasets are available through Socrata API^2^. In this study, we retrieved the NSOD datasets using Socrata API using the Python^3^ programming language. We wrote a command-line tool to fetch the datasets and performed an exploratory analysis to understand the datasets. At the time of this research, there are 669 datasets in 28 categories, of which 77.8% are archived datasets, and 22.2% are currently active. Most of the datasets were created between April 2016 and June 2016 and gradually updated each year. The majority of collected datasets were in the English language. Around 79.7% of the datasets have Nova Scotia province defined as their region, while 20.3% datasets have missing values in region metadata. The top categories of datasets are “Environment and Energy” (58), “Health and Wellness” (52), “Population and Demographics” (48), “Business and Industry” (37) and “Education” (32). Overall, we found 21 disease-related datasets in the “Health and Wellness” category by searching the NSOD web portal using the “disease” keyword. Each NSOD dataset has a metadata section and an observation section that includes the statistical observations. The datasets had the same structure in terms of the name and number of attributes (e.g., year, disease name, number of cases). Figure 1 shows the structure of disease-related datasets. There were 13 observations in each dataset, including statistical information about disease cases in the Nova Scotia province between 2005 and 2017.

## Methodology

A knowledge graph construction process can be performed based on the following steps: 1) Knowledge acquisition to collect semi-structured data from an API, 2) Knowledge extraction to extract entities and their relationships, 3) Knowledge fusion to construct an ontology, assigning entities and relationships and interlink entities to external ontologies and datasets, and 4) Knowledge storage to create knowledge graph in a triple store. To generate a knowledge graph for the NSOD disease datasets, we transform the collected datasets to RDF using a multi-dimensional data model, a custom ontology, semantic rules, and an interlinking process. The following subsections will describe the steps in detail.

### Data model

**Fig. 2:**
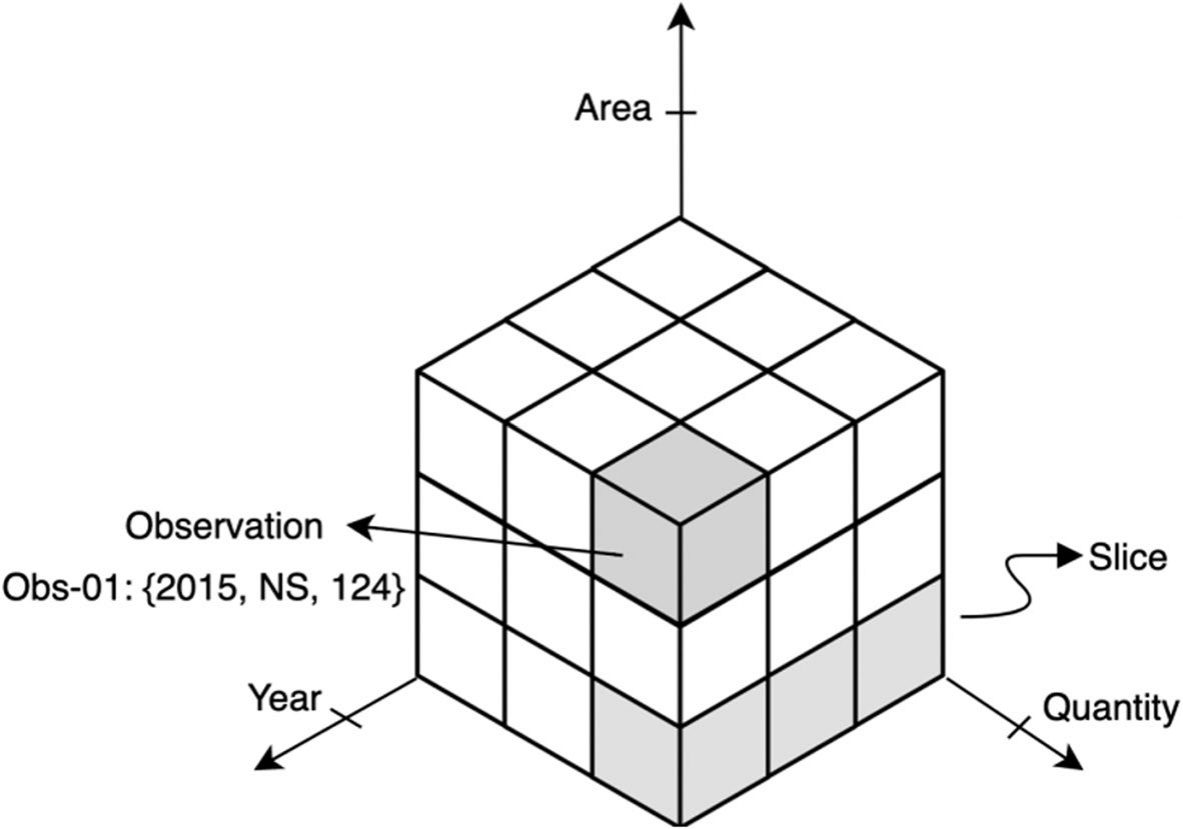
An example of observation in an open statistical dataset [4]

The metadata of each NSOD dataset consists of information about that dataset, such as name, publisher, publication date, category, department, etc., which can be transformed to RDF using VoiD [12], DCMI^4^, DCAT^5^, and RDFS vocabularies. The observation of an NSOD dataset includes a collection of dimensions, measures, and attributes that can be shown as Data Structure Definition (DSD). Figure 2 shows an observation example in an NSOD dataset.

To model the multi-dimensional NSOD datasets, the RDF Data Cube vocabulary^6^ is used based on the W3C recommendation [13]. The RDF Cube allows publishers to integrate and slice across their datasets [14]. This enables the representation of the statistical data in standard RDF format and publishes the data conforming to the principles of linked data [15]. Slices are frequently useful to group subsets of observations within a dataset. For instance, we can group all the observations about a given region or category in a dataset. 

### Ontology

To the best of our knowledge, no existing ontologies can be re-used based on the nature of the NSOD datasets. However, we re-use a current data model for describing multi-dimensional data (RDF Cube vocabularies), an external disease ontology, and the best practice vocabularies such as Statistical Data and Metadata eXchange (SDMX) to develop a custom ontology for the disease-related datasets of NSOD. The datasets were coded as entities with distinct data structure definitions, slices and observations.

**Fig. 3:**
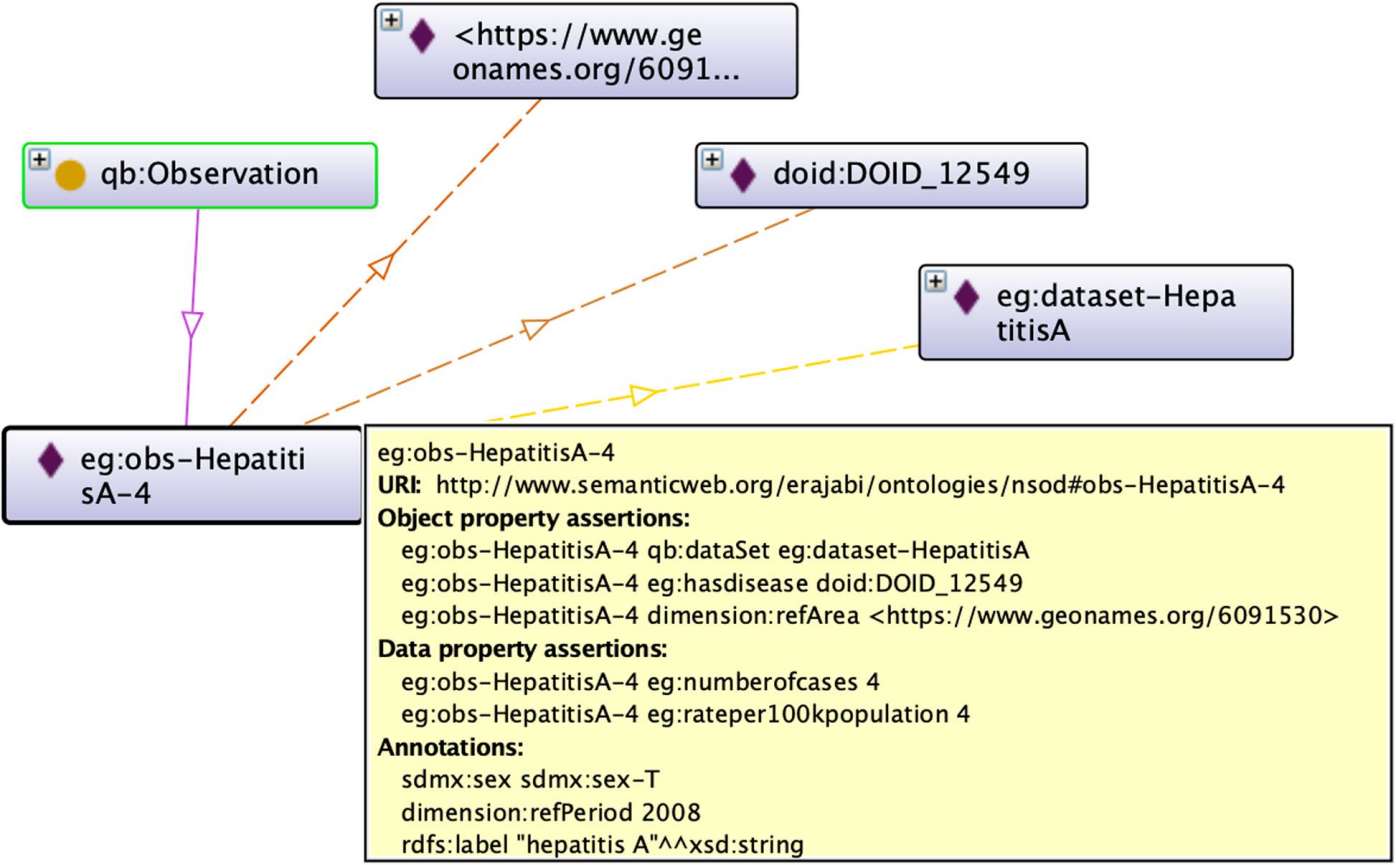
An observation based on the defined ontology

All the datasets in the ontology are instances of class *DataSet* and the nomenclature used for datasets is *“dataset-dataset_name”*. Each dataset has one associated data structure definition (*qb:DataStructureDefintion*), which defines the dataset’s dimensions, measures, and attributes linked with *DataSet* by *structure* property. The dimensions, measures and attributes are linked with the data structure definition by properties *dimension*, *measure*, and *attribute*, respectively. Also, class *qb:Slice* and *ObservationGroup* are used to group observations by one or more dimensions. Each slice is linked to the data structure definition using *sliceKey* property. The observations are attached to a dataset by the *observation* property and the respective slices by the *observationGroup* property. Figure 3 illustrates a sample observation based on the defined ontology. Table 1 also shows the prefixes used in the ontology.

**Table 1:** Re-used vocabularies

Vocabulary	Prefix	Usage
RDF Cube	http://purl.org/linked-data/cube#	Multi-dimensional, observations
Dublin Core	http://purl.org/dc/terms	Metadata of datasets
DOID	http://purl.obolibrary.org/obo/doid#	The disease ontology
GeoNames	http://www.geonames.org/ontology#	Geographical information
SDMX	http://purl.org/linked-data/sdmx/2009/code#	Dimensions and measures
SWRL	http://swrl.stanford.edu/ontologies/3.3/swrla.owl#	Semantic rules
VoiD	http://rdfs.org/ns/void#	Dataset description

### Interlinking datasets to external ontology and datasets

We use an external ontology, Disease Ontology^7^ to enrich the knowledge graph with domain knowledge. We link the NSOD diseases to the disease ontology based on the cosine similarity between the disease names. According to this interlinking process, we enrich the disease information by its parent (super-class) diseases and enable users to search the knowledge graph based on the disease direct super-classes (e.g., viral disease). We also use Geonames^8^ to represent regional dimension information instead of literal. This allows the addition of semantics to statistical data in case the other regional datasets (other provincial datasets) are joined to the knowledge graph.

As the DBpedia knowledge graph^9^ includes a broad scope of entities covering different areas of disease knowledge, we also connect the disease names of an NSOD dataset to this knowledge graph. To perform this, we use Python to search for each disease name in DBpedia using SPARQL via its SPARQL endpoint and connect each observation to the DBpedia source using *owl:sameAs* vocabulary. For example, the disease *Giardiasis* is linked to http://dbpedia.org/resource/Giardiasis.

### Rules

Complex formal semantics in a knowledge graph allows a reasoner to infer the relationship between data items in different datasets [16]. This step is carried out to add more meaning to the knowledge graph and links the entities together using an additional semantic layer. The Semantic Web Rule Language (SWRL^10^), an example of a Rule Markup Language, is used to standardize the publishing and sharing of inference rules. As a proof of concept, we design an SWRL rule to infer the transitive relationship of diseases in a dataset using Protégé^11^ rule engine. This implies that if an observation *x* includes a disease *y*, which is a form of disease *z* in the disease ontology, then the graph will infer that observation *x* includes the disease *z* implicitly. The rule states that:

$$\begin{aligned} hasDisease(?x, ?y)\, \wedge \, \textit{doid:is}\_a(?y, ?z) \implies hasDisease(?x, ?z) \end{aligned}$$

Another semantic rule example is related to the observations with the highest number of cases for a particular disease. Based on the current number of cases of each disease in the Nova Scotia province, we considered 1,000 disease cases per 100,000 population to be high in the province. Those observations can be defined by the following rule:

$$\begin{aligned} Observation(?obs) \wedge numberOfCases(?obs,&?n) \wedge \textit{swrlb:greaterThan}(?n, 1000) \\&\implies HighDiseaseCases(?obs) \end{aligned}$$

### Transformation process

The structural metadata about the dimensions and measures of the NSOD datasets are generally different. We develop a configuration setting to specify the dimensions and measures of each dataset in case other datasets with various dimensions and measures are added. This allows semi-automatic updating of the graph with input data and makes the datasets semantically connected to the external ontologies and the Linked Open Data cloud. For example, several disease datasets had *number of cases* property that could be used as one predicate (*eg:numberOfCases*) across the knowledge graph.

In the transformation process, we use the Dublin Core Metadata [17], the most widely used metadata schema, to describe the metadata elements of datasets such as published date, dataset title, subject or category, source, contributor, etc. The corresponding elements of each observation are mapped to RDF triples based on the vocabularies mentioned in Table 2).

**Table 2:** Mapping vocabularies

Section	Element	Mapping voacbulary
Metadata	Dataset licence	dct:license
Metadata	Dataset language	dct:language
Metadata	Department	:department
Metadata	Dataset description	rdfs:comment
Metadata	Dataset keyword	dcat:keyword
Metadata	Dataset suject	dcat:theme
Observation	Year of observation	sdmx-dimension:refPeriod
Observation	Region of observation	sdmx-dimension:refArea
Observation	Number of cases for each disease	:numberOfCases
Observation	An observation belongs to a disease	:hasDisease
Observation	Case rate per 100,000 population	:rateper100kpopulation
Observation	Gender in observation	sdmx:sex
Observation	Geolocation of dataset	dct:spatial

### Knowledge graph constructor

**Fig. 4:**
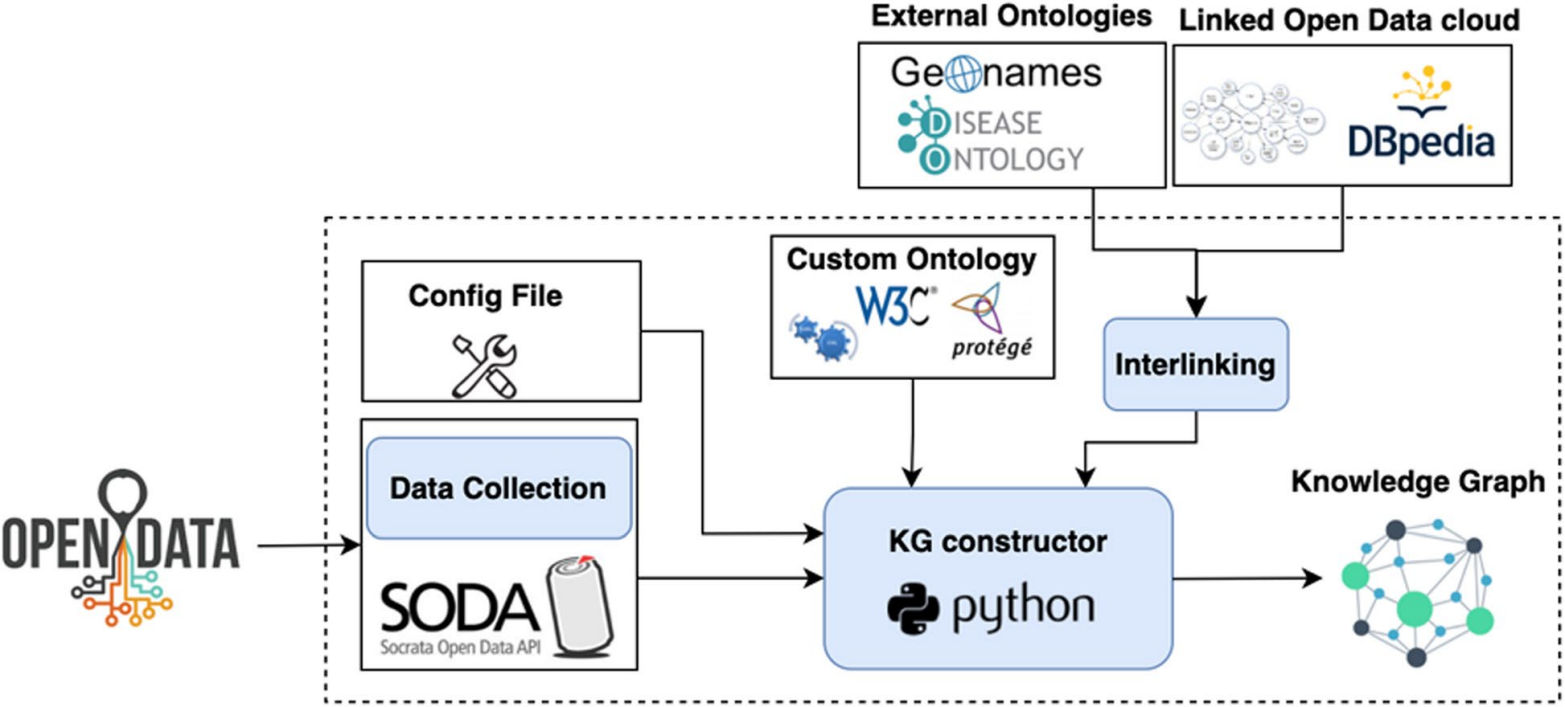
Knowledge graph construction process

The knowledge graph constructor is the main component of the knowledge graph construction process (see Fig. 4). It connects various parts of the system by collecting data from different sources, transforming them into a unified multi-dimensional model based on the W3C standards, interlinking them with external ontologies, and translating the defined rules to enable semantic reasoning over the knowledge graph. Eventually, the datasets are added to the graph as observations, ensuring that they conform to prescribed metadata, structure, and Semantic Web protocols. We wrote a Python program to construct the knowledge graph which is available at https://github.com/erajabi/Nova_Scotia_Open_Data.

### Queries

We use the built-in SPARQL^12^ tab in Protégé to pose a set of designed queries against the knowledge graph, which cannot be explicitly expressed through linkage. We design the questions with the help of Nova Scotia health stakeholders considering the semantic rules developed in Rules section in the knowledge graph. For example, some diseases re the sub-classes of the infectious disease class in the disease ontology, and we use *rdfs:subClassOf* propertyto retrieve the results. The queries are outlined below.

**Fig. 5:**
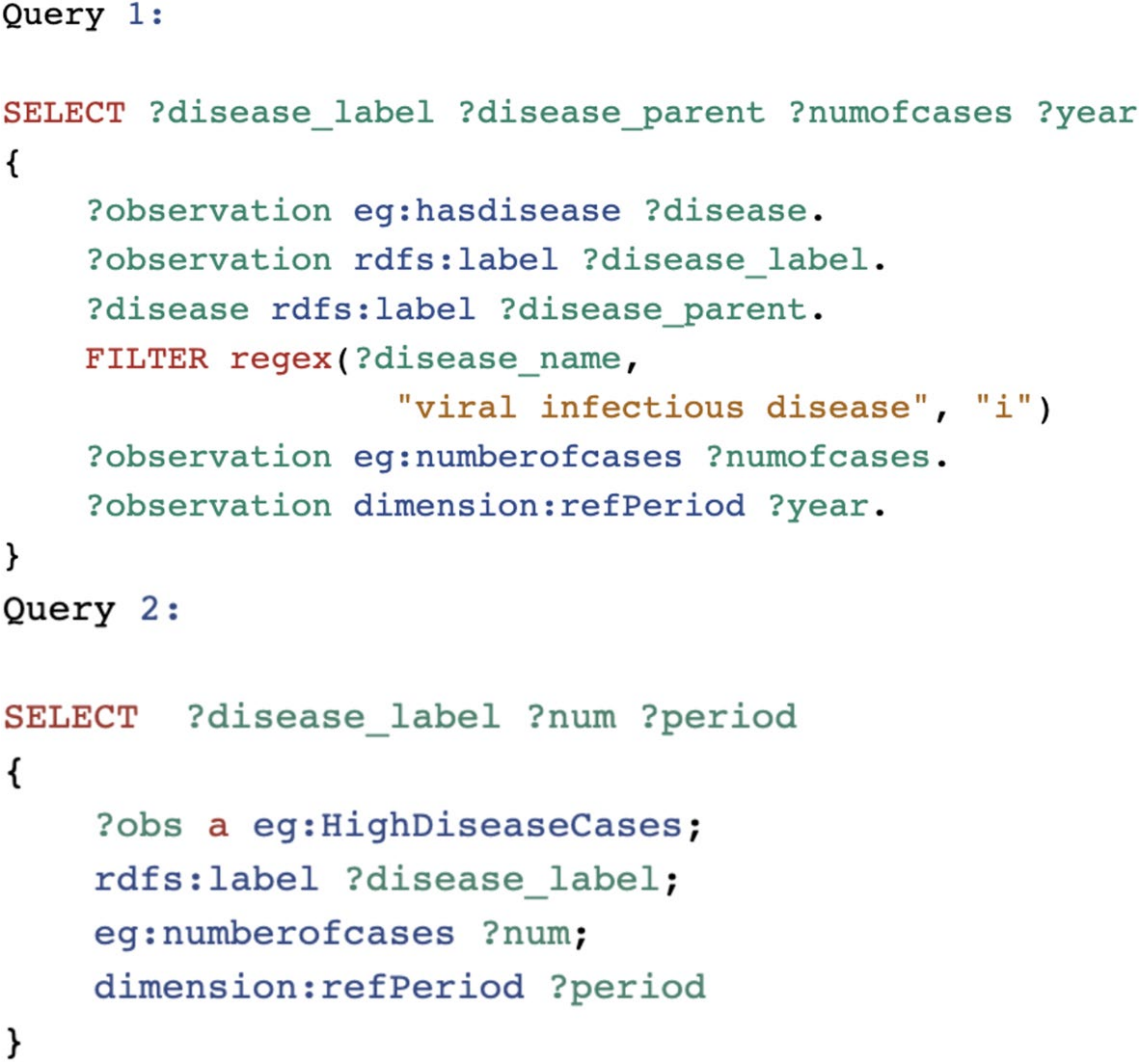
The designed queries. An online SPARQL editor was used to improve the readability of the SPARQL Queries

Figure 5 shows two queries we define along with the sample results. In both queries, we leverage the rules that we defined before.

*Query 1: List of viral infectious diseases along with their number of cases in Nova Scotia in different years.*

In this query, we use *doid:is_a* relationship rule to identify all the disease classified as “viral infectious diseases”.

*Query 2: List of viral infectious diseases with a high number of cases (more than 1,000 cases) in Nova Scotia in 2017.*

In this question, we use the *HighDiseaseCases* class to infer the results based upon the rule defined in Rules section.

### Knowledge graph

The final knowledge graph included 2,883 triples with 24 classes, 23 object properties, and two data properties. All 21 disease datasets were successfully transformed into the knowledge graph, with a total of 252 observations. Each observation includes several dimensions such as gender (*sdmx:sex*), observation year (*dimension:refPeriod*), and area of observation (*dimension:refArea*). It also contains a few measures such as disease rate per 100k population of disease (*eg:rateper100kpopulation*) and a number of disease cases (*numberofcases*). Additionally, an observation has disease information (*eg:hasDisease*) and disease label (*rdfs:label*) properties, which has been connected to the DBpedia knowledge graph using *owl:sameAs* property. The knowledge graph is publicly available at Zenodo under Creative Commons Universal Public Domain Dedication (CC0 1.0)^13^ license.

## Discussion

Knowledge graphs represent factual knowledge as entities and their relationships using a graph data model [18]. They include metadata along with taxonomies of entities and their relationships. Knowledge graphs can be used for knowledge retrieval, question-answering, creating unified-data access points, and recommendation systems. In this study, we constructed a knowledge graph for query-answering by creating a data pipeline to collect data from the NSOD web portal and mapping them to a knowledge graph based on the W3C standards. We demonstrated the integration of disease-related datasets of an open government data portal, as there are many datasets in the NSOD web portal in various domains. As illustrated in Methodology section, one of the applications of such a knowledge graph is linking the open government datasets across a province or a country. For example, each Canadian province publishes its statistical datasets in an open data portal, while they are not connected to each other. Furthermore, the datasets are not linked to the other datasets in other provinces. Currently, the relationship between the entities has not been specified in the open government data. Creating a knowledge graph from statistical datasets for provinces or countries facilitates data integration and query answering, and can be used as a single data access point.

As a use case, having the NSOD knowledge graph can answer some questions like: “Did we have an increase in the number of respiratory disease cases in Nova Scotia in recent years”? Given that the hierarchical relationships between the entities have been defined in the knowledge graph using the Disease Ontology, all diseases that are a subclass of respiratory disease are retrieved. Similarly, larger knowledge graphs, including disease-related datasets across the country, can be used to answer a query like: “What is the number of viral disease cases in the Nova Scotia and Alberta provinces in 2022?”. This question can be answered using the dimensional properties of the knowledge graph (e.g., the region name, year, and hierarchical relationships of each observation).

Publishing statistical and biomedical data in knowledge graphs has many advantages. Knowledge graphs have made a qualitative leap and effected a real revolution in knowledge representation due to their underlying structure, which underpins a better comprehension, reasoning, and interpretation of knowledge for both humans and machines [19]. As knowledge graphs present a common framework for knowledge representation [20], they have recently become an increasingly popular research direction and gained significant attention from both industry and academia in scenarios that require exploiting diverse, dynamic, large-scale collections of data [21, 22]. They continue to be used as the main means of solving many real-life problems in various domains [19] and consequently can support many biomedical applications with a particular focus on machine learning approaches, as it is easier to link data from different knowledge graphs and make predictions within genomic, pharmaceutical, and clinical domains [23]. Existing non-proprietary technologies in the Semantic Web, such as logical rules (SWRL), constraints (Shex, SHACL), and instantiation (JSON-LD) allows adding new semantic layers to data and bring more insights.

The advantage of using RDF graphs has motivated the data migration from other formats to RDF for some years [24–26]. Although some tools have been proposed to facilitate the data migration [27–29], it remains time-consuming, and error-prone [19]. Therefore, we argue that sharing knowledge about migrating real-world datasets to RDF is valuable in helping data engineers reach a consensus on best practices. Additionally, the NSOD knowledge graph represents an effort made by public authorities worldwide to publish information about their public services to facilitate discovery, and use by citizens and businesses [30]. Some examples of governmental data published as a knowledge graph are the datasets about public procurement [31, 32], missing persons [26, 33], policies [34]. healthcare [35], and diseases such as cancer [36].

As mentioned in [37], there are a few challenges that data ecosystems face on their way to adding a knowledge layer to datasets and making them smarter. Mappings between the datasets and adding an ontology describe the meaning of the datasets and enhance data transparency. Although the data mapping and transformation process in this study has been performed using a software program, there is a hindrance in completing the automatic construction of a knowledge graph. Identifying the disease-related datasets was done manually, making the knowledge graph construction process semi-automatic. Most disease-related datasets in the NSOD portal contain the same dimensions (such as year, area, and disease name), though this might not be true for all the datasets. The lack of descriptive metadata that explicitly enlist each dataset’s dimensions, measures, and attributes was another significant hurdle toward achieving complete automation. Alternatively, the lack of a vocabulary that supports properties (e.g., ex:numberOfCases) that convey this information is another issue that prevents us from addressing it in a standardized manner.

We also did not leverage a natural language processing model to transform the user-defined questions to SPARQL. A text-to-SPARQL approach similar to [38] could be used in the knowledge graph construction process to facilitate the question answering.

## Conclusion

In this study, we introduced the NSOD disease knowledge graph as a unique data access point for the disease-related datasets of the NSOD portal. We leveraged the Semantic Web standards, such as RDF Cube and SWRL, to create the knowledge graph. During the exploratory analysis of the extracted datasets, we noticed that different provincial open data portals across Canada publish datasets with the same structure and related topics. A Linked Data strategy, similar to what we described in this article, can be used to build a SPARQL endpoint (e.g., in the Canada Open Data portal^14^) to connect similar open statistical datasets across a country and facilitate query answering for data consumers and the linked open data community.

## Data Availability

Not applicable.

## Footnotes

1 https://data.novascotia.ca

2 https://dev.socrata.com/

3 https://www.python.org

4 https://dublincore.org

5 https://www.w3.org/TR/vocab-dcat-2/

6 https://www.w3.org/TR/vocab-data-cube/

7 https://disease-ontology.org/

8 https://www.geonames.org

9 https://dbpedia.org/sparql

10 https://www.w3.org/Submission/SWRL/

11 https://protege.stanford.edu

12 https://doi.org/10.5281/zenodo.7539374

13 https://creativecommons.org/publicdomain/zero/1.0/

14 https://open.canada.ca/
